# How adolescent depressive symptoms are associated with premature mortality: Behavioral and socioeconomic pathways across the life course

**DOI:** 10.1007/s00787-026-03103-6

**Published:** 2026-06-25

**Authors:** NaKyung Nam, Hyewon Son, Jinho Kim

**Affiliations:** 1 Department of Health Policy and Management, Korea University, Room 367, B-dong Hana-Science Building 145 Anam-ro, Seongbuk-gu, Seoul, Republic of Korea; 2 Interdisciplinary Program in Precision Public Health, Korea University, Seoul, Republic of Korea; 3 Department of Sociology, The University of Texas at Austin, Austin, TX USA; 4 Center for Demography of Health and Aging, University of Wisconsin- Madison, Madison, WI USA

**Keywords:** Adolescent depressive symptoms, Premature mortality, Young adulthood, Health behaviors, Socioeconomic status, Mediation

## Abstract

Premature mortality in young adulthood has become a growing public health concern in the United States, yet it remains unclear whether vulnerability to early death originates as early as adolescence. Although depressive symptoms are highly prevalent during adolescence and linked to long-term health and socioeconomic disadvantages, their prospective association with premature mortality remains poorly understood. This study examined whether depressive symptoms experienced during adolescence predict premature mortality in young adulthood and whether health behaviors and socioeconomic status in early adulthood help explain this association. Data were drawn from Waves I and III of the National Longitudinal Study of Adolescent to Adult Health, along with the Mortality Outcomes Surveillance System established in Wave V (*N* = 14,320). Cox proportional hazards models estimated the association between adolescent depressive symptoms and premature mortality, and cause-specific Cox proportional hazards models evaluated deaths due to disease, suicide, and external causes. Mediation analyses tested health behaviors and socioeconomic factors as mechanisms. Higher adolescent depressive symptoms were associated with increased risk of all-cause premature mortality in young adulthood. Cause-specific models showed that this association was concentrated in deaths due to external causes, with no significant associations for disease-related deaths or suicide. Cigarette smoking, marijuana use, lower educational attainment, and reduced employment in early adulthood accounted for part of the association for all-cause mortality. For external-cause mortality, however, health behaviors—specifically cigarette smoking and marijuana use—emerged as the prominent mediating pathways. Findings suggest that adolescent depressive symptoms may be associated with an increased vulnerability to premature mortality, in part through behavioral and socioeconomic pathways that emerge in early adulthood. Strengthening early mental health identification and support could reduce long-term disparities in survival.

**Supplementary Information:**

The online version contains supplementary material available at https://doi.org/10.1007/s00787-026-03103-6.

## Introduction

Premature mortality in young adulthood remains a pressing and persistent public health concern in the United States. Mortality rates among individuals in their 20 s, 30 s, and 40 s have plateaued or even risen in recent decades, fueled in part by increases in suicide, drug overdose, injury, and chronic illness [1–3]. Identifying early-life predictors of these deaths is therefore critical for determining who is most vulnerable and for informing prevention strategies well before mortality risk becomes clinically apparent. One factor that warrants particular attention is adolescent depressive symptoms. Adolescence is marked by heightened emotional reactivity and the emergence of many mental health conditions, and depressive symptoms during this period have been linked to enduring consequences for health and well-being across the life course [4, 5]. Depressive symptoms are also highly prevalent among U.S. adolescents and represent not only clinical depressive disorders but a broader continuum of subclinical emotional difficulties that often remain undetected and untreated [6]. If emotional distress experienced during adolescence contributes to vulnerability to death decades later, the etiology of premature mortality may originate far earlier in development than is typically assumed.

Despite growing concern about the long-term consequences of adolescent mental health, only a small number of longitudinal studies suggest that depression may elevate the risk of early death in adulthood [7–11], and evidence specifically assessing whether depressive symptoms in adolescence predict premature mortality in young adulthood remains scarce. Most research on depression and mortality has instead examined depression measured in adulthood, consistently documenting elevated risks of death from chronic disease, suicide, and injury [12, 13]. However, this literature largely reflects depressive disorders assessed in midlife or later adulthood, long after individuals have established behavioral patterns, social roles, and physical health profiles. Consequently, it is unclear whether depression identified much earlier in the life course carries similar implications. Adolescent depressive symptoms constitute a distinct developmental phenomenon—preceding most adult pathways and encompassing a broad spectrum of subclinical emotional distress that rarely receives treatment yet affects roughly one in five U.S. adolescents annually [14, 15]. Clarifying whether they predict premature mortality would therefore determine whether vulnerability to early death originates much earlier than currently recognized.

This long-term association is rooted in the ‘sensitive period’ of health development, a stage where adolescent experiences exert a disproportionate and lasting impact on lifelong trajectories [16, 17]. During this period, elevated depressive symptoms may be linked to alterations in biobehavioral systems, whereby early psychological distress becomes biologically embedded and predisposes individuals to physiological vulnerabilities that increase mortality risk in later years [16]. Furthermore, as adolescence represents a critical timing in lives for transitions into adult roles, depressive symptoms may be associated with subsequent disruptions in educational and occupational attainment, thereby initiating a cumulative process of socioeconomic disadvantage [17, 18]. Consequently, the adolescent window serves as a critical intersection where psychological distress and socioeconomic contexts interact to define the foundational parameters for premature mortality.

Building on this framework, there may be two major pathways through which adolescent depressive symptoms contribute to premature mortality in young adulthood. The first pertains to health behaviors. A substantial body of work shows that adolescents reporting elevated depressive symptoms are more likely to initiate cigarette smoking earlier, smoke more frequently, and continue smoking into adulthood; they also tend to engage in heavier and more frequent alcohol consumption, including binge drinking, and report greater marijuana and illegal drug use compared with their peers [19, 20]. Depressive symptoms are further associated with heightened risk of obesity, often linked to dysregulated eating patterns, emotional eating, and reduced physical activity [21, 22]. These behavioral profiles align with well-established predictors of elevated morbidity and mortality risk, given that smoking, heavy alcohol use, marijuana use, illicit drug use, and obesity substantially increase the likelihood of chronic disease, overdose, injury, and other external causes of death [23, 24]. Taken together, these patterns suggest that health behaviors represent a plausible pathway through which adolescent depressive symptoms may prospectively increase vulnerability to premature mortality.

The second pathway relates to socioeconomic status in early adulthood. Depressive symptoms in adolescence have been shown to disrupt educational trajectories, including lower academic performance, reduced school engagement, and decreased likelihood of high school completion [25–27]. Educational setbacks during adolescence can compound over time, shaping later opportunities in the labor market and contributing to lower employment rates, unstable job tenure, and reduced earnings in adulthood [28]. Depressive symptoms are also linked to reduced likelihood of obtaining or maintaining health insurance coverage, which in turn limits access to preventive and clinical services and increases financial barriers to care [29, 30]. These socioeconomic disadvantages reflect well-documented social gradients in mortality, whereby educational attainment, employment opportunities, income, and healthcare access play central roles in determining long-term survival [31, 32]. Thus, socioeconomic status in early adulthood offers another plausible mechanism through which adolescent depressive symptoms may prospectively contribute to premature mortality.

The present study investigates whether depressive symptoms experienced during adolescence prospectively predict premature mortality in young adulthood using data from the National Longitudinal Study of Adolescent to Adult Health (Add Health), a nationally representative cohort followed for nearly three decades (1994–2023). We examine both all-cause and cause-specific mortality and incorporate a comprehensive set of individual- and family-level covariates measured at baseline. In addition, we assess whether health behaviors and socioeconomic status in early adulthood help to explain the association between adolescent depressive symptoms and mortality risk, thereby evaluating two theoretically grounded life-course mechanisms. By integrating long-term prospective data, mortality follow-up through midlife, and a multidimensional consideration of behavioral and socioeconomic pathways, this study advances the literature on adolescent mental health and population health by clarifying whether vulnerability to premature death emerges as early as adolescence and identifying key processes that may account for this link.

## Data and method

### Data

This study utilized data from the National Longitudinal Study of Adolescent to Adult Health (Add Health), a nationally representative cohort of U.S. adolescents enrolled in grades 7–12 during the 1994–95 school year (Wave I). Participants were followed prospectively across four subsequent waves—1996 (Wave II), 2001–2002 (Wave III), 2008–2009 (Wave IV), and 2016–2018 (Wave V)—allowing observation of developmental and health trajectories from adolescence into adulthood. The original Wave I cohort included 20,745 individuals, with follow-up response rates ranging from 77% to 89% [33]. Add Health captures a broad range of social, environmental, and health-related characteristics at the individual, family, school, and neighborhood levels.

To monitor mortality as participants entered their 30 s and 40 s, Add Health established the Mortality Outcomes Surveillance System at Wave V. Extensive tracing procedures—such as locating updated addresses and conducting in-person field visits—were used to identify respondents lost to follow-up. Individuals determined to be deceased or potentially deceased were submitted to the National Death Index (NDI) using identifying information (e.g., name, sex, date of birth, and Social Security number). Matches were evaluated using a validated scoring algorithm, and cases with a 1:1 match were confirmed as deaths. This multistage process enabled vital status determination for every member of the original cohort, with mortality data updated through December 31, 2023 [34].

For the present analysis, data from Waves I and III of the In-Home Survey were combined with data from the Mortality Outcomes Surveillance System established in Wave V. Adolescent depressive symptoms were measured at Wave I, and mechanism variables were drawn from Wave III. Among 14,322 respondents with complete participation across these two waves with Add Health longitudinal survey weights, two individuals who died before Wave III were excluded due to missing data on mediating variables, resulting in a final analytic sample of 14,320 (473 confirmed deaths). Missing covariate data were addressed using multiple imputation by chained equations in Stata 18.0, generating 10 imputed datasets to reduce potential bias and strengthen statistical precision. Written informed consent was obtained from all participants (or from parents/guardians for minors) during data collection. Because this secondary analysis was conducted using de-identified public-use data, it was deemed exempt from full review by an institutional review board.

### Measures

#### Dependent variable

This study assessed both all-cause and cause-specific mortality, with vital status verified through December 31, 2023. Importantly, all deaths identified within the analytic sample occurred before age 50, allowing the outcome to be conceptualized as premature mortality during early to mid-adulthood. Causes of death were grouped into three broad categories based on the International Classification of Diseases, 10th Revision (ICD-10): (1) disease-related deaths (e.g., disorders of the nervous, circulatory, respiratory, or digestive systems); (2) external causes (e.g., transport accidents, accidental poisoning, or assault); and (3) suicide (e.g., intentional self-poisoning or self-inflicted injury). The survival time was calculated as the number of months between the Wave I interview and either the month of death or December 2023 for those who were still alive at follow-up.

#### Independent variable

Adolescent depressive symptoms were assessed at Wave I using a 9-item short form of the Center for Epidemiologic Studies Depression (CES-D) scale. Respondents indicated how often each statement had been true for them during the past week: (1) being bothered by things that usually do not bother them; (2) being unable to shake the blues even with support from others; (3) feeling just as good as other people; (4) having difficulty concentrating; (5) feeling depressed; (6) feeling too tired to carry out activities; (7) enjoying life; (8) feeling sad; and (9) feeling disliked by others. Response categories ranged from 0 (“never or rarely”) to 3 (“most or all of the time”). A composite CES-D score was created by summing responses across the nine items and then standardizing the total score to facilitate interpretation. The scale demonstrated good internal consistency (Cronbach’s α = 0.79).

#### Mediating variable

Mediating variables were drawn from Wave III and included two domains: health behaviors (cigarette smoking, binge drinking, marijuana use, illicit drug use, and obesity status) and socioeconomic status (high school completion, employment status, personal earnings, and health insurance coverage). Current cigarette smoking was coded as 1 if respondents reported smoking on at least one day during the past 30 days. Binge drinking was coded as 1 when individuals indicated consuming five or more alcoholic drinks in a row at least once per week during the past 12 months. Marijuana use was coded as 1 if respondents reported using marijuana at least once in the past 30 days. Illicit drug use was coded as 1 if respondents reported using any illegal drugs (e.g., LSD, PCP, ecstasy, mushrooms, speed, ice, heroin, or pills) at least once during the past 30 days. Obesity status was defined as a BMI ≥ 30, calculated as weight in kilograms divided by height in meters squared. High school completion was based on self-reported highest level of formal education completed. Current employment was coded as 1 if respondents reported working for pay at least 10 h per week. Personal earnings were based on self-reported annual pre-tax income in categorical brackets; each bracket was converted to its midpoint value and log-transformed to reduce right skew. Health insurance coverage was coded as 1 if respondents indicated having any type of insurance plan.

#### Control variable

To account for potential confounding, the analysis controlled for a wide range of individual- and family-level characteristics that may be associated with both adolescent depressive symptoms and subsequent mortality risk. Unless otherwise noted, all covariates were assessed at baseline (Wave I). Individual-level controls included gender, age, grade in school, race/ethnicity (White, Black, Hispanic, or other), foreign-born status, firstborn status, and cognitive ability measured using the standardized Peabody Picture Vocabulary Test (PVT) score. Family-level covariates included maternal education, household income, co-residence with both biological parents, number of siblings, rural residence, maternal ever smoking, and indicators of emotional, physical, and sexual abuse.

Maternal education was based on the respondent-reported highest grade completed by the mother and coded as total years of schooling. Household income was derived from parent reports to the question: “How much total income, before taxes, did your family receive in 1994? Include your own income, the income of everyone else in your household, and income from welfare benefits, dividends, and all other sources.” Income was reported in categorical increments (up to $100,000) and treated as a continuous measure. Co-residence with both biological parents was coded as 1 if the adolescent reported living with both their biological mother and biological father at the time of the Wave I interview. Number of siblings was based on the adolescent’s response to the question: “How many children have your biological parents had together?” Rural residence was defined using the interviewer’s assessment of the respondent’s immediate residential area.

Maternal ever smoking was coded as 1 if the respondent reported that the resident mother had ever smoked cigarettes. Emotional abuse, physical abuse, and sexual abuse were assessed at Wave I using retrospective self-reports of experiences occurring before age 18. Respondents were asked how often, prior to their 18th birthday: (1) a parent or other adult caregiver said things that really hurt their feelings or made them feel unwanted or unloved (emotional abuse); (2) a parent or adult caregiver hit them with a fist, kicked them, or threw them down on the floor, into a wall, or down stairs (physical abuse); and (3) a parent or other adult caregiver touched them in a sexual way, forced them to touch the adult in a sexual way, or forced them to have sexual relations (sexual abuse). Each abuse measure was coded as a dichotomous indicator (1 = experienced at least once before age 18; 0 = never experienced).

### Analytic strategy

We estimated the association between adolescent depressive symptoms and mortality using a series of Cox proportional hazards regression models. Our primary specification modeled time to death from any cause as the outcome:

$$\:{h}_{i}\left(t\right)={h}_{0}\left(t\right)\:exp\:(\beta\:{ADS}_{i}+{{X}}_{{i}}\gamma\:)$$

where $\:{h}_{i}\left(t\right)$ denotes the hazard of death for individual *i* at time *t*, conditional on survival up to that time. The baseline hazard function $\:{h}_{0}\left(t\right)$ represents the underlying risk of death shared across individuals. The independent variable, $\:{ADS}_{i}$, is adolescent depressive symptoms, and ${{X}}_{{i}}$ is a vector of baseline covariates. The coefficient $\:\beta\:$ indicates the log–hazard ratio associated with adolescent depressive symptoms, such that positive values reflect elevated mortality risk.

To further distinguish variation in mortality risks attributable to different underlying causes, we extended the analysis to cause-specific mortality. Cause-specific Cox proportional hazards models were estimated separately for three mutually exclusive categories of death: disease-related mortality, externally caused mortality, and suicide. Following the Add Health analytic guidelines [35], all Cox proportional hazards models incorporated the full complex survey design specification: the longitudinal survey weight to account for unequal probability of selection, oversampling, and non-response; the primary sampling unit (PSUSCID, school identification variable) to adjust for clustering of students within schools; and the post-stratification variable (REGION, census region) to account for stratification. A subpopulation indicator was used to define the analytic sample, ensuring that all primary sampling units contributed to the variance estimation even when individual observations fell outside the analytic subsample [35]. Models were estimated in Stata using the survey-adjusted Cox regression command with multiple imputation (mi estimate: svy, subpop(): stcox).

To assess the robustness of our findings, we conducted E-value analyses to estimate the minimum strength of association that an unobserved confounder would need to have with both the exposure and the outcome to fully explain away the observed associations [36]. As a sensitivity check, we re-estimated models using family-level cluster-robust standard errors to account for sibling clustering. Results were virtually identical to those from the primary models (e.g., SE = 0.0159 vs. 0.0160 for depressive symptoms). The four-way decomposition procedure, as implemented in the med4way command in Stata, does not natively support clustered standard errors; while the sampling weight (GSWGT3_2) is applied to produce correct point estimates, the command does not accommodate the svy: prefix or the vce(cluster) option for specifying the primary sampling unit and strata variables.

To investigate the extent to which health behaviors and socioeconomic status in early adulthood mediated the association between adolescent depressive symptoms and premature mortality, we conducted a 4-way decomposition [37]. Using the med4way command in Stata, this approach decomposes the total effect of the exposure (adolescent depressive symptoms) on the outcome (mortality) into four distinct components: (1) the controlled direct effect (CDE), representing the effect of depressive symptoms on mortality net of the mediators; (2) the pure indirect effect (PIE), representing the portion of the effect mediated through the mediator, independent of exposure–mediator interaction; (3) the mediated interaction (INTmed), capturing the portion of the effect attributable to the interaction between adolescent depressive symptoms and the mediators; and (4) the reference interaction (INTref), representing interaction effects independent of mediation [38]. By separating these components, the 4-way decomposition provides a more comprehensive characterization of mediation processes than traditional methods, allowing us to assess not only whether mechanisms explain part of the association but also whether the strength of such mechanisms differs across levels of depressive symptoms. All mediation models incorporated the same set of baseline covariates included in the main Cox regression models.

## Results

Table 1 presents unweighted descriptive statistics for the analytic sample of 14,320 individuals. Approximately 3% of respondents in the analytic sample were confirmed deceased by December 2023. During 4,842,663 person-months of follow-up (maximum 359 months, approximately 30 years), 473 deaths were observed, including 228 deaths from disease (48.2%), 181 from external causes (38.3%), 46 from suicide (9.7%), and 18 from other causes (3.8%). The mean age at death was 36.41 years (SD = 6.47; range: 20–48; median = 38), underscoring the premature nature of mortality in this cohort—all deaths occurred before age 50. Baseline age at Wave I did not differ significantly between survivors (M = 16.1, SD = 1.7) and decedents (M = 16.1, SD = 1.8; t = − 0.04, *p* = 0.97). Males comprised 58.1% of decedents compared to 46.9% of survivors (χ² = 23.3, *p* < 0.001).

**Table 1:** Unweighted Descriptive Statistics, Add Health

Variable	Wave	Mean/Prop.	SD	Min	Max
Dependent variable					
Mortality status (Deceased)	2023/12	3.3%		0.000	1.000
Disease-related		48.2%^*^			
External causes		38.3%^*^			
Suicide		9.7%^*^			
Other Causes		3.8%^*^			
Independent variable					
Depressive symptoms	Wave I	5.943	4.286	0.000	27.000
Mechanism variable					
Health behaviors					
Cigarette smoking	Wave III	20.9%		0.000	1.000
Binge drinking	Wave III	46.9%		0.000	1.000
Marijuana use	Wave III	21.1%		0.000	1.000
Illicit drug use	Wave III	4.2%		0.000	1.000
Obesity status	Wave III	19.4%		0.000	1.000
Socioeconomic status					
High school completion	Wave III	82.6%		0.000	1.000
Current employment status	Wave III	69.7%		0.000	1.000
Log earnings	Wave III	7.426	3.532	0.000	13.617
Health insurance coverage	Wave III	50.5%		0.000	1.000
Control variable					
Age	Wave I	16.115	1.726	12.000	21.000
Male	Wave I	47.2%		0.000	1.000
White	Wave I	54.1%		0.000	1.000
Black	Wave I	22.0%		0.000	1.000
Hispanic	Wave I	15.6%		0.000	1.000
Asian	Wave I	6.9%		0.000	1.000
Others	Wave I	1.4%		0.000	1.000
Foreign-born status	Wave I	8.3%		0.000	1.000
First-born child	Wave I	50.3%		0.000	1.000
Standardized PVT score	Wave I	0.056	0.967	−5.648	3.055
Maternal education	Wave I	13.151	2.432	0.000	17.000
Family income	Wave I	0.462	0.520	0.000	9.990
Co-residence of both biological parents	Wave I	54.0%		0.000	1.000
Number of siblings	Wave I	1.607	1.445	0.000	13.000
Rural status	Wave I	26.0%		0.000	1.000
Maternal ever smoking	Wave I	46.3%		0.000	1.000
Emotional abuse	Wave I	46.3%		0.000	1.000
Physical abuse	Wave I	18.0%		0.000	1.000
Sexual abuse	Wave I	8.2%		0.000	1.000
Observations		14,320			

Summary statistics contain imputed values. *Add Health* National Longitudinal Study of Adolescent to Adult Health, *PVT* Picture Vocabulary Test. In the estimation models, the standardized depressive symptoms score was used to facilitate interpretation of effect sizes

* Percentages reflect the distribution among 473 confirmed deaths

The key independent variable, depressive symptoms measured at Wave I, had a mean of 5.94 (SD = 4.29; range: 0–27). Mechanism variables assessed at Wave III reveal substantial variability in early-adulthood health behaviors and socioeconomic conditions: 21% reported current cigarette smoking, 47% engaged in weekly binge drinking, 21% used marijuana, 4% reported illicit drug use in the past 30 days, and 19% met the clinical threshold for obesity. Socioeconomic characteristics indicate that 83% had completed high school, 70% were currently employed, and 51% had health insurance coverage, while log earnings averaged 7.43. Baseline control variables measured at Wave I reflect the demographic and family profile of the cohort: the mean age was 16.12 years (SD = 1.73), 47% were male, and the majority identified as White (54%), followed by Black (22%), Hispanic (16%), and Asian (7%). Approximately half were firstborn children (50%), and 54% resided with both biological parents.

Table 2 presents results from Cox proportional hazards regression models estimating the association between adolescent depressive symptoms and all-cause mortality in early to mid-adulthood. Model 1 includes no covariates beyond the key independent variable and shows that a one-standard-deviation increase in adolescent depressive symptoms is associated with a 21.7% increase in the hazard of death (HR = 1.217, 95% CI [1.073, 1.381]). Model 2 adjusts for individual-level characteristics, and although the magnitude of the association is slightly reduced, adolescent depressive symptoms remain a significant predictor of mortality (HR = 1.224, 95% CI [1.068, 1.402]). Model 3 additionally incorporates family-level variables, and the association persists, indicating that even after accounting for both individual and family characteristics at baseline, a one-standard-deviation increase in adolescent depressive symptoms is linked to an 17.5% higher risk of all-cause mortality (HR = 1.175, 95% CI [1.019, 1.356]).

**Table 2:** Cox proportional hazards models of the association between adolescent depressive symptoms and all-cause mortality

	(1)	(2)	(3)
	All-cause mortality	All-cause mortality	All-cause mortality
	HR [95% CI]	HR [95% CI]	HR [95% CI]
Individual-level covariates	No	Yes	Yes
Family-level covariates	No	No	Yes
Adolescent depressive symptoms	1.217^**^	1.224^**^	1.175^*^
	[1.073,1.381]	[1.068,1.402]	[1.019,1.356]
Age		0.990	0.991
		[0.904,1.085]	[0.905,1.085]
Male		1.687^***^	1.704^***^
		[1.288,2.209]	[1.297,2.239]
Black		1.002	0.914
		[0.717,1.399]	[0.646,1.294]
Hispanic		0.743	0.688
		[0.437,1.264]	[0.397,1.191]
Asian		0.254^**^	0.263^**^
		[0.101,0.639]	[0.102,0.674]
Others		1.542	1.464
		[0.659,3.609]	[0.662,3.240]
Foreign-born status		0.567	0.561
		[0.228,1.410]	[0.223,1.412]
First-born child		1.008	0.927
		[0.762,1.334]	[0.678,1.270]
Standardized PVT score		0.769**	0.795^**^
		[0.665,0.889]	[0.675,0.936]
Maternal education			0.977
			[0.918,1.039]
Family income			0.653
			[0.360,1.186]
Co-residence of both biological parents			0.834
			[0.631,1.102]
Number of siblings			0.977
			[0.888,1.076]
Rural status			1.032
			[0.783,1.360]
Maternal ever smoking			0.940
			[0.723,1.220]
Emotional abuse			1.270
			[0.921,1.752]
Physical abuse			1.229
			[0.842,1.795]
Sexual abuse			0.853
			[0.461,1.578]
*N*	14,320	14,320	14,320

In all estimation models, the full Add Health complex survey design was incorporated, including longitudinal survey weights (GSWGT3_2), clustering by school (PSUSCID), and post-stratification by census region (REGION), with subpopulation analysis. Hazard ratios (HRs) with 95% confidence intervals in brackets are reported. Individual-level covariates include age, gender, race/ethnicity, foreign-born status, first-born child, and standardized PVT score. Family-level covariates include maternal education, family income, co-residence of both biological parents, number of siblings, rural status, maternal ever smoking, emotional abuse, physical abuse, and sexual abuse

+ *p* < 0.1; * *p* < 0.05; ** *p* < 0.01; *** *p* < 0.001

Table 3 reports results from cause-specific Cox proportional hazards models models estimating the association between adolescent depressive symptoms and cause-specific mortality. After adjusting for all individual- and family-level covariates, depressive symptoms in adolescence were not significantly associated with mortality from disease-related causes (HR = 1.034, 95% CI [0.816, 1.312]) or suicide (HR = 1.407, 95% CI [0.983, 2.015]). However, a significant association emerged for mortality from external causes, such that a one-standard-deviation increase in adolescent depressive symptoms was linked to a 29.6% higher hazard of death due to external causes (HR = 1.296, 95% CI [1.046, 1.604]). While this pattern underscores a pronounced concentration of risk in externally caused deaths, the results should be interpreted with caution given the relatively small number of deaths in each cause-specific category—especially suicide—which may limit statistical power and precision.

**Table 3:** Cause-specific Cox proportional hazards models of the association between adolescent depressive symptoms and cause-specific mortality

	(1)	(2)	(3)
	Mortality (Disease-related cause)	Mortality (External cause)	Mortality (Suicide)
	HR [95% CI]	HR [95% CI]	HR [95% CI]
Individual-level covariates	Yes	Yes	Yes
Family-level covariates	Yes	Yes	Yes
Adolescent depressive symptoms	1.034	1.296^*^	1.407^+^
	[0.816,1.312]	[1.046,1.604]	[0.983,2.015]
Age	1.051	0.945	0.886
	[0.911,1.213]	[0.834,1.071]	[0.656,1.197]
Male	1.141	2.374^***^	4.213^**^
	[0.785,1.658]	[1.557,3.621]	[1.790,9.914]
Black	1.513^+^	0.473*	0.657
	[0.969,2.363]	[0.262,0.852]	[0.243,1.778]
Hispanic	0.687	0.631	1.335
	[0.300,1.571]	[0.249,1.596]	[0.428,4.161]
Asian	0.530	0.018^***^	0.187
	[0.183,1.536]	[0.003,0.113]	[0.017,2.076]
Others	2.906*	0.438	1.107
	[1.121,7.533]	[0.109,1.757]	[0.145,8.461]
Foreign-born status	0.650	0.168^+^	0.770
	[0.184,2.293]	[0.021,1.364]	[0.128,4.630]
First-born child	1.117	0.882	0.714
	[0.683,1.826]	[0.580,1.341]	[0.275,1.852]
Standardized PVT score	0.819	0.768^*^	0.870
	[0.630,1.065]	[0.606,0.974]	[0.636,1.190]
Maternal education	0.963	0.984	1.048
	[0.881,1.052]	[0.884,1.096]	[0.842,1.302]
Family income	0.800	0.637	0.185
	[0.417,1.533]	[0.222,1.829]	[0.016,2.139]
Co-residence of both biological parents	0.748	0.832	1.370
	[0.495,1.131]	[0.519,1.333]	[0.557,3.371]
Number of siblings	0.960	1.037	0.968
	[0.822,1.120]	[0.900,1.195]	[0.774,1.211]
Rural status	1.256	0.858	0.959
	[0.852,1.849]	[0.563,1.310]	[0.404,2.277]
Maternal ever smoking	0.616^**^	1.714^*^	0.623
	[0.427,0.888]	[1.097,2.679]	[0.289,1.340]
Emotional abuse	1.268	1.261	1.570
	[0.817,1.968]	[0.778,2.044]	[0.575,4.284]
Physical abuse	1.181	1.335	0.961
	[0.688,2.026]	[0.762,2.341]	[0.290,3.182]
Sexual abuse	0.823	0.760	1.724
	[0.389,1.740]	[0.277,2.084]	[0.510,5.822]
*N*	14,320	14,320	14,320

In all estimation models, the full Add Health complex survey design was incorporated, including longitudinal survey weights (GSWGT3_2), clustering by school (PSUSCID), and post-stratification by census region (REGION), with subpopulation analysis. Hazard ratios (HRs) with 95% confidence intervals in brackets are reported. Individual-level covariates include age, gender, race/ethnicity, foreign-born status, first-born child, and standardized PVT score. Family-level covariates include maternal education, family income, co-residence of both biological parents, number of siblings, rural status, maternal ever smoking, emotional abuse, physical abuse, and sexual abuse

+ *p* < 0.1; * *p* < 0.05; ** *p* < 0.01; *** *p* < 0.001

To assess the robustness of our findings to unmeasured confounding, we conducted E-value sensitivity analyses and tests of nonlinearity. For the association between standardized adolescent depressive symptoms and all-cause mortality (HR = 1.175), the E-value was 1.628 (E-value for the confidence interval limit: 1.158). For external-cause mortality (HR = 1.296), the E-value was 1.915 (CI limit: 1.265). These values suggest that an unmeasured confounder would need to be associated with both depressive symptoms and mortality by a risk ratio of at least 1.628 (all-cause) or 1.915 (external cause), above and beyond measured covariates, to fully explain the observed associations. We also tested for nonlinearity by including a quadratic term for depression in the fully adjusted models (Table S1 in the online supplementary file); the quadratic terms were not significant for either all-cause (HR = 1.040, 95% CI [0.974, 1.110]) or external-cause mortality (HR = 0.974, 95% CI [0.877, 1.082]), indicating a linear relationship. Quartile analyses further supported this pattern, though the highest quartile showed a markedly elevated risk of external-cause mortality compared to the lowest (HR = 2.260, 95% CI [1.283, 3.981]), suggesting that the risk is most pronounced among those with the highest levels of depressive symptoms. Intermediate quartile estimates (Q2, Q3) were smaller in magnitude and non-significant, which likely reflects reduced precision due to categorization. The continuous model therefore provides the most reliable interpretation, as it retains more information and yields more stable estimates.

Tables 4 and 5 present the results of four-way decomposition analyses examining nine potential mediators of the depression–mortality association. For all-cause mortality (Table 4), the pure indirect effects (PIE) through cigarette smoking (coef. = 0.018, *p* < 0.05), marijuana use (coef. = 0.017, *p* < 0.05), obesity (coef. = 0.006, *p* < 0.05), high school completion (coef. = 0.025, *p* < 0.05), and employment status (coef. = 0.006, *p* < 0.05) were statistically significant. High school completion showed the largest proportion mediated (16.0%), followed by cigarette smoking (10.8%) and marijuana use (9.0%). For external-cause mortality (Table 5), significant PIEs were observed for cigarette smoking (coef. = 0.029, *p* < 0.05) and marijuana use (coef. = 0.032, *p* < 0.05), with proportions mediated of 6.9% and 12.2%, respectively. Notably, across both outcomes, the controlled direct effect consistently accounted for the largest share of the total effect, and the interaction components (INT_ref and INT_med) were generally non-significant, indicating that the mediation pattern is driven mainly by the pure indirect pathway rather than through exposure-mediator interaction. These results indicate that while the majority of the depression–mortality association is explained by direct pathways, health behaviors and socioeconomic factors partially mediate the relationship—most notably through high school completion (16.0%), marijuana use (9.0–12.2%), and cigarette smoking (6.9–10.8%).

**Table 4:** Mediation analysis of the association between adolescent depressive symptoms and all-cause mortality

Mediator	Total Effect		CDE		INT_ref		INT_med		PIE		% Med
	Coef.	SE	Coef.	SE	Coef.	SE	Coef.	SE	Coef.	SE	
Cigarette smoking	0.181^*^	0.080	0.150	0.082	0.012	0.039	0.002	0.006	0.018^*^	0.006	10.8%
Binge drinking	0.176^*^	0.081	0.233^*^	0.110	−0.056	0.068	−0.001	0.002	0.001	0.002	−0.3%
Marijuana	0.181^*^	0.081	0.171^*^	0.085	−0.007	0.038	−0.001	0.005	0.017^*^	0.005	9.0%
Illicit drug use	0.176^*^	0.080	0.167^*^	0.079	0.005	0.018	0.001	0.003	0.004	0.002	2.7%
Obesity status	0.179^*^	0.081	0.192^*^	0.090	−0.018	0.035	−0.001	0.002	0.006^*^	0.003	2.9%
High school completion	0.177^*^	0.081	0.244	0.218	−0.096	0.196	0.003	0.007	0.025^*^	0.007	16.0%
Current employment status	0.181^*^	0.080	0.110	0.160	0.065	0.123	−0.001	0.002	0.006^*^	0.003	2.9%
Log earnings	0.180^*^	0.080	0.077	0.203	0.101	0.176	−0.001	0.002	0.003	0.002	1.4%
Health insurance coverage	0.172^*^	0.081	0.103	0.110	0.069	0.074	−0.000	0.001	0.000	0.001	0.0%

*CDE* controlled direct effect, *INT_ref* reference interaction, *INT_med* mediated interaction, *PIE* pure indirect effect. % Mediated = overall proportion mediated. In all estimation models, the full Add Health complex survey design was incorporated, including longitudinal survey weights (GSWGT3_2), clustering by school (PSUSCID), and post-stratification by census region (REGION), with subpopulation analysis. All models adjusted for age, gender, race/ethnicity, foreign-born status, first-born child, standardized PVT score, maternal education, family income, co-residence of both biological parents, number of siblings, rural status, maternal ever smoking, emotional abuse, physical abuse, and sexual abuse

* *p* < 0.05

**Table 5:** Mediation analysis of the association between adolescent depressive symptoms and external-cause mortality

Mediator	Total Effect		CDE		INT_ref		INT_med		PIE		% Med
	Coef.	SE	Coef.	SE	Coef.	SE	Coef.	SE	Coef.	SE	
Cigarette smoking	0.350^*^	0.126	0.358^*^	0.117	−0.032	0.066	−0.005	0.010	0.029^*^	0.009	6.9%
Binge drinking	0.301^*^	0.129	0.268	0.145	0.029	0.107	0.001	0.002	0.004	0.003	1.4%
Marijuana	0.302^*^	0.123	0.227^*^	0.103	0.038	0.070	0.005	0.009	0.032^*^	0.009	12.2%
Illicit drug use	0.301^*^	0.127	0.279^*^	0.118	0.012	0.040	0.002	0.006	0.009	0.005	3.7%
Obesity status	0.292^*^	0.124	0.396^*^	0.142	−0.103	0.045	−0.006	0.003	0.005	0.004	−0.6%
High school completion	0.307^*^	0.134	0.313	0.276	−0.020	0.251	0.001	0.009	0.014	0.009	4.6%
Current employment status	0.297^*^	0.130	0.331	0.268	−0.043	0.194	0.001	0.003	0.008	0.004	2.9%
Log earnings	0.297^*^	0.129	0.436	0.354	−0.143	0.293	0.002	0.003	0.002	0.003	1.1%
Health insurance coverage	0.286^*^	0.130	0.312	0.201	−0.026	0.114	0.000	0.000	0.000	0.002	0.0%

*CDE* controlled direct effect, *INT_ref* reference interaction, *INT_med* mediated interaction, *PIE* pure indirect effect. % Mediated = overall proportion mediated. In all estimation models, the full Add Health complex survey design was incorporated, including longitudinal survey weights (GSWGT3_2), clustering by school (PSUSCID), and post-stratification by census region (REGION), with subpopulation analysis. All models adjusted for age, gender, race/ethnicity, foreign-born status, first-born child, standardized PVT score, maternal education, family income, co-residence of both biological parents, number of siblings, rural status, maternal ever smoking, emotional abuse, physical abuse, and sexual abuse

* *p* < 0.05

To evaluate potential overlap among mediators, we examined pairwise correlations across all mediating variables (Table S2 in the online supplementary file). Correlations were generally low to moderate in magnitude. The strongest associations were observed between marijuana use and other illicit drug use (*r* = 0.326), log earnings and current employment (*r* = 0.309), and marijuana use and binge drinking (*r* = 0.286). All remaining correlations were smaller. Although these results suggest some conceptual clustering within behavioral and socioeconomic domains, the correlations do not indicate substantial multicollinearity. Because mediators were evaluated separately within the four-way decomposition framework, the estimated proportions mediated represent pathway-specific effects and should not be interpreted as additive across mediators.

## Discussion

This study examined whether depressive symptoms experienced during adolescence prospectively predict premature mortality in young adulthood. Using nearly three decades of nationally representative longitudinal data (1994–2023), we found that higher levels of adolescent depressive symptoms were associated with an elevated risk of all-cause mortality in young adulthood, even after accounting for a wide range of individual and family characteristics measured at baseline. Cause-specific Cox proportional hazards models revealed that this association was concentrated in deaths due to external causes—such as accidents, overdose, and assault—rather than disease-related deaths. These findings extend prior research linking depression in adulthood to increased mortality risk (Cuijpers & Smit, 2002; Walker et al., 2015) by demonstrating that vulnerability to early death may originate much earlier in the life course. They also build on a small number of longitudinal studies suggesting that adolescent depression may predict mortality later in adulthood [7–11] by showing that elevated mortality risk is already evident by young adulthood, well before midlife health decline typically begins.

With respect to suicide, we observed a trend-level positive association, though this remained below conventional statistical significance thresholds. This pattern could be interpreted in light of several considerations. First, the relatively small number of suicide deaths may have limited statistical power and the precision of estimates. Second, our exposure measure, CES-D, captures subclinical depressive symptomatology during adolescence rather than acute clinical episodes of major depression. Prior research suggests that mild or subclinical depressive symptoms show weak or null associations with suicide risk, whereas moderate-to-severe clinical depression demonstrates substantially elevated risk [39]. Third, ICD-10 classification constraints mean that some deaths of despair—particularly drug overdoses—are coded as accidental poisonings within the external causes category. Since these deaths may overlap with suicidal intent, this classification may attenuate the suicide-specific estimate while contributing to the robust association observed for external causes.

The pathway analyses provide additional insight into how depressive symptoms in adolescence may be linked to premature mortality in young adulthood. Consistent with life-course models identifying behavioral and socioeconomic antecedents to early death [11], health behaviors in early adulthood—particularly cigarette smoking and marijuana use—accounted for a meaningful share of the association between adolescent depressive symptoms and mortality risk. These findings align with prior work showing that emotional distress during adolescence can predict early adoption and sustained engagement in health-compromising behaviors [19, 20]. Socioeconomic factors also emerged as a salient pathway: lower educational attainment and reduced likelihood of employment partially accounted for the association, echoing evidence that adolescent mental health difficulties can disrupt educational transitions and labor market trajectories in ways that accumulate into long-term survival disadvantages [40, 41]. Taken together, these results suggest that the link between adolescent depressive symptoms and premature mortality may not be fully explained solely by biological vulnerability or psychiatric severity, but also through social and behavioral processes that characterize opportunity structures and exposure to risk over the early life course.

Notably, these behavioral pathways were particularly salient for external-cause mortality, with cigarette smoking and marijuana use mediating 6.9% and 12.2% of this specific association, respectively. While socioeconomic and behavioral factors both played significant mediating roles in overall premature mortality, the risk of dying from external causes—such as accidents, overdoses, and violence—was predominantly channeled through these health-compromising behaviors. This divergence suggests that adolescent depressive symptoms may drive young people toward substance use as a form of self-medication or coping, which in turn acutely elevates their vulnerability to fatal incidents in early adulthood through impaired judgment, increased impulsivity, or heightened exposure to hazardous situations [42, 43]. While socioeconomic disadvantages, such as disrupted education and unemployment, remain critical mechanisms for overall health decline, the acute threat of external-cause mortality during this developmental stage appears more directly triggered by the behavioral sequelae of early emotional distress [44, 45].

These findings carry important conceptual implications for understanding how early-life mental health predicts long-term population health. By demonstrating that depressive symptoms measured in adolescence—well before chronic disease onset or midlife social stratification—predict premature mortality, this study reinforces life-course perspectives that emphasize the developmental origins of adult health and survival (Ben-Shlomo & Kuh, 2002; Viner et al., 2015). The concentration of risk in deaths due to external causes also suggests that adolescent depressive symptoms may function less as a marker of underlying biological deterioration and more as an indicator of vulnerability to environments and circumstances that elevate exposure to hazards during critical transitional periods [46, 47]. Furthermore, the distinct explanatory roles of health behaviors and socioeconomic status underscore the importance of understanding the acute risks of maladaptive coping mechanisms, particularly substance use, within the broader context of socioeconomic disadvantage, rather than treating mental health as a purely individual or clinical phenomenon [48, 49]. These insights highlight the need for theoretical frameworks that recognize adolescent mental health not merely as an outcome of early experiences but as a potential turning point linked to differences in life trajectories with profound implications for survival [50].

A few limitations should be considered when interpreting these findings. First, although the study leverages nearly three decades of prospective data, depressive symptoms were measured only at a single time point in adolescence, which limits the ability to distinguish between transient versus chronic depressive trajectories and to capture symptom fluctuations across development. Second, cause-specific mortality analyses were based on a relatively small number of deaths in some categories, particularly suicide, and results for these endpoints should therefore be interpreted with caution. Specifically, the external causes category aggregates diverse mechanisms (transport accidents, accidental poisoning, assault), and the Add Health mortality data do not provide sufficient code granularity to distinguish these causes. Future research using larger samples and detailed ICD-10 subcodes could explore diverse mortality mechanisms within external causes, including growing public health concerns such as drug overdose deaths, to clarify how different pathways link adolescent depression to premature mortality.

Third, while the Cox proportional hazards models incorporate the full complex survey design specification—including sampling weights, clustering by school (PSUSCID), and post-stratification by census region (REGION)—the four-way mediation decomposition analyses using the med4way command in Stata do not fully account for these design features. Although sampling weights are applied to the med4way models to produce correct point estimates, the command does not natively support the svy: prefix, the vce(cluster) option, or subpopulation analysis. Consequently, the standard errors from the mediation analyses may be slightly underestimated. Future replication studies should consider alternative software packages (e.g., Mplus) that are capable of integrating causal mediation analysis with the full complex survey design specification.

The findings of this study carry important implications for public health and policy. Demonstrating that depressive symptoms in adolescence are associated with premature mortality in young adulthood underscores the need to view youth mental health as a population health priority rather than solely an individual clinical concern [51, 52]. Policies that expand early identification and timely treatment of depressive symptoms in school and community settings—including routine mental health screening, school-based counseling, and low-barrier referral pathways—may help interrupt life-course processes that heighten mortality risk [53–55]. Because the pathway analyses indicate that subsequent health behaviors and socioeconomic disadvantage play a role, prevention efforts should extend beyond symptom management to address both fatal behavioral risks and broader socioeconomic contexts [56]. Specifically, to mitigate the external-cause mortality risks driven by health behaviors, mental health support should be integrated with substance use prevention and harm-reduction strategies [57, 58]. Moreover, strategies such as comprehensive school mental health programs, investment in dropout-prevention and educational support, and workplace training and transition programs tailored to youth with mental health challenges have shown promise in reducing inequalities in employment, income, and access to healthcare [59, 60]. In this way, integrating mental health support into educational, health, and social policy systems could reduce not only psychological burden but also long-term disparities in survival.

In conclusion, this study provides evidence that depressive symptoms experienced during adolescence are prospectively associated with an elevated risk of premature mortality in young adulthood, particularly deaths due to external causes. These findings extend existing research by demonstrating that vulnerability to early death may originate much earlier in the life course than previously recognized and by identifying health behaviors and socioeconomic disadvantage as potential explanatory pathways. Although further research is needed to refine causal mechanisms and evaluate how changing social, economic, and mental health contexts predict these patterns across cohorts, the present results underscore the importance of early detection and sustained support for adolescent mental health as a strategy for promoting long-term population health. To build on these findings, future longitudinal studies should track how contemporary societal dynamics—including the opioid crisis and increasing social isolation—predict mortality trajectories in more recent cohorts. Additionally, integrating macro-level structural variables, notably neighborhood deprivation or social safety net policies, will be essential to further elucidate these complex relationships. Interventions that effectively address depressive symptoms during adolescence—and the distinct behavioral and socioeconomic sequelae that often follow—may not only reduce psychological burden but also are associated with narrowing inequalities in survival across the life span.

## Electronic Supplementary Material

Below is the link to the electronic supplementary material.

Supplementary Material 1 (DOCX 15.2 KB)

## Data Availability

The data that support the findings of this study are available from the Carolina Population Center. Restrictions apply to the availability of these data, which were used under license for this study. Data are available at [https://data.cpc.unc.edu/](https://data.cpc.unc.edu) with the permission of the Carolina Population Center.
