# Supplementary material for: How adolescent depressive symptoms are associated with premature mortality: Behavioral and socioeconomic pathways across the life course
Source: Eur Child Adolesc Psychiatry. 2026 Jun 25;35(11):3449–61. doi: 10.1007/s00787-026-03103-6 (PMC13710569; doi:10.1007/s00787-026-03103-6)
Supplement: Supplementary file 1 [file 787_2026_3103_MOESM1_ESM.docx]

# Online supplementary file

## Table S1. Tests of nonlinearity in the depression-mortality association

|  | (1) | (2) | (3) | (4) |
| --- | --- | --- | --- | --- |
|  | All-cause mortality | All-cause mortality | Mortality  (External cause) | Mortality  (External cause) |
|  | HR | HR | HR | HR |
|  | [95% CI] | [95% CI] | [95% CI] | [95% CI] |
| Individual-level covariates | Yes | Yes | Yes | Yes |
| Family-level covariates | Yes | Yes | Yes | Yes |
| **Quadratic term models** |  |  |  |  |
| Std CES-D (linear) | 1.111 |  | 1.349^*^ |  |
|  | [0.925,1.335] |  | [1.021,1.783] |  |
| Std CES-D squared | 1.040 |  | 0.974 |  |
|  | [0.962,1.125] |  | [0.869,1.091] |  |
| **Quartile models (Ref: Q1 (lowest))** |  |  |  |  |
| Q2 |  | 0.962 |  | 1.660 |
|  |  | [0.601,1.541] |  | [0.849,3.247] |
| Q3 |  | 1.005 |  | 0.984 |
|  |  | [0.680,1.486] |  | [0.504,1.921] |
| Q4 (highest) |  | 1.351 |  | 2.260^**^ |
|  |  | [0.941,1.941] |  | [1.251,4.082] |
| *N* | 14320 | 14320 | 14320 | 14320 |

*Note.* In all estimation models, the full Add Health complex survey design was incorporated, including longitudinal survey weights (GSWGT3_2), clustering by school (PSUSCID), and post-stratification by census region (REGION), with subpopulation analysis. Hazard ratios (HRs) with 95% confidence intervals in brackets are reported. Individual-level covariates include age, gender, race/ethnicity, foreign-born status, first-born child, and standardized PVT score. Family-level covariates include maternal education, family income, co-residence of both biological parents, number of siblings, rural status, maternal ever smoking, emotional abuse, physical abuse, and sexual abuse.

+ *p* < 0.1; * *p* < 0.05; ** *p* < 0.01; *** *p* < 0.001

## Table S2. Pairwise correlations among potential mediators

|  | (1) | (2) | (3) | (4) | (5) | (6) | (7) | (8) | (9) |
| --- | --- | --- | --- | --- | --- | --- | --- | --- | --- |
| (1) Cigarette smoking | 1.0 |  |  |  |  |  |  |  |  |
| (2) Binge drinking | 0.158 | 1.0 |  |  |  |  |  |  |  |
| (3) Marijuana use | 0.239 | 0.286 | 1.0 |  |  |  |  |  |  |
| (4) Illicit drug use | 0.133 | 0.156 | 0.326 | 1.0 |  |  |  |  |  |
| (5) Obesity status | -0.000 | -0.052 | -0.044 | -0.035 | 1.0 |  |  |  |  |
| (6) High school diploma | -0.167 | 0.039 | -0.061 | -0.013 | -0.049 | 1.0 |  |  |  |
| (7) Current employment | 0.023 | 0.077 | -0.006 | -0.006 | -0.013 | 0.093 | 1.0 |  |  |
| (8) Log earnings | 0.028 | 0.114 | 0.033 | 0.031 | -0.026 | 0.103 | 0.309 | 1.0 |  |
| (9) Health insurance coverage | -0.037 | -0.051 | -0.110 | -0.067 | 0.044 | 0.013 | 0.198 | 0.101 | 1.0 |

*Note.* N = 14,320. All mediators measured at Wave III.
